# Administration of genetic expression by multi-task proteins and long-range action

**DOI:** 10.3389/fgene.2013.00052

**Published:** 2013-04-10

**Authors:** Michèle Amouyal

**Affiliations:** Centre National de la Recherche Scientifique-Interactions à DistanceParis, France

In eukaryotes, a gene must be switched on or off at a given time of development, in a given tissue, in a given environment. To meet these specific obligations and accommodate their diversity, the gene mobilizes genetic elements all over the genome and factors which are not necessarily transcription-specific. The 10 articles of this book highlight the latest advances in the topic, focusing on the extraordinary adaptability of some of these proteins and their unexpected spectrum of competencies.

Historically, enhancers were the first elements found to act at-a-distance from the gene, as exhaustively reviewed by Palstra and Grosveld (2012), starting from the β-globin locus, a model for cellular differentiation. It is now well evidenced that these enhancers contact promoters with looping of in-between chromatin.

Other elements, such as insulators, assist this process. A prototype insulator is CTCF. At the β-globin locus (Palstra and Grosveld, 2012), the Igf2/H19 parent-of-origin imprinted locus (Singh et al., 2012), or at the immunoglobulin heavy chain antibodies locus (Birshtein, 2012), CTCF confers a basal folding to the genome, creating the proximity necessary for the productive contacts. CTCF also blocks RNA elongation at pause sites, sharing this feature with prokaryotic factors in addition to looping. Last, insulators are implicated in epigenetic regulation, counteract the spread of heterochromatin, decide of chromatin composition.

This 3-D genomic architecture allows to direct enhancer action from one gene to another, to coordinate expression of several genes or genetic loci simultaneously, to couple and control genes within one unit or several processes with the same factors, if necessary, sometimes delineating eukaryotic equivalents of prokaryotic operons. This structural scaffold provides strengthened, yet dynamic, interactions, stable enough to allow other contacts to take place, to resist to moving cellular tensions, even possibly to cell division.

However, the frontier between the different classes: (1) promoters/proximal elements, (2) enhancers, (3) insulators, are not clearly defined. The cell actually makes use of any element to ensure proper genetic expression. Hence, some promoters (for RNA polymerases II or III) and basal/proximal elements of the transcriptional machinery of initiation such as TFIIIC, indifferently act as insulators, as reported in (Amouyal, 2012; Holwerda and de Laat, 2012; Palstra and Grosveld, 2012). The LCR enhancer, of which the deletion leads to thalassemia, conversely illustrates this ambiguity. Once thought to only act by counteracting the spread of heterochromatin like some insulators, it also operates like classical enhancers by contacting promoter with specific factors (EKLF, GATA-1, FOG-1) and looping.

In addition, some factors perform other tasks than genetic expression, using the same structural device at the molecular level. Thus, according to cell cycle progression, but always by ensuring chromosomal cohesion, cohesin is either structurally involved in (1) sister chromatids cohesion/DNA damage repair, or in (2) gene transcription with chromosomal looping at several loci. Multi-functionality here explains the diversity of phenotypes in cohesinopathies due to defective cohesin, from Roberts to Cornelia de Lange syndromes, with all intermediates (Horsfield et al., 2012).

The extensive utilization by the cell of a peculiar skill for different applications is not restricted to DNA loopers. Thus PARP1 transcription factor (reviewed by Beneke, 2012) has the capacity to synthesize poly(ADP)ribose and to transfer it either covalently or non-covalently to other proteins. Addition of this polymeric sugar to CTCF presumably improves chromosomal looping by providing a dimerization interface and by stabilizing CTCF DNA-binding at several loci. Cancer marks a defective process. But PARP1 is also part of the basal RNA polymerase II machinery (as TFIIC), both a positive and negative cofactor of transcription, and mediates the response to DNA damage with the same tool. Thus, it loosens chromatin structure for the access of appropriate factors by the simple interaction of the poly(ADP-ribose) with histones.

In the same vein, CTCF regulates coding mRNAs as well as non-coding RNAs, in the same field of tumor suppression, control of cell cycle and proliferation, including embryonic stem cell differentiation for RNA regulators (Saito and Saito, 2012), which nicely corroborates the way CTCF acts in one case, anticipates it in the second one.

The cell also makes use of long-range action at different levels to assist gene expression. First thought to be confined to enhancer-promoter interaction, it has been extended with insulators to the structuring of a whole locus, and at an upper level, to genome-/cell-wide organization, by means of the same factors/auxiliaries of transcription, as if “he who can the least, can the most.”

Holwerda and de Laat (2012) tackle the question of gene positioning within the nucleus in this context. The new technologies (Hi-C, *lac* operators tethered to lamina,…) indicate a susceptibility to gene silencing close to the nuclear periphery or at the heart of chromosome territories. Out of these locations, up to 1 Mb domains of active chromatin are enriched at their border with insulators (CTCF, tRNAs, SINEs,…) instigating, at least contributing to this partitioning. These technically difficult and fully progressing *in vivo* researches are somewhat in line with earlier studies related to the nuclear matrix and genome attachment defined by chemical treatments.

Transition from DNA looping to high-order genomic organization is not surprising as the same elements (DNA repeats and protein apt to oligomerize in the simplest case) lead to intra-chromosomal looping, chromosomal clustering and condensation when reproduced, inter-chromosomal interactions, coating with arrays of tandem repeats.

In fact, genomic repetition is as common as enhancer occurrence and is extremely susceptible to genome rearrangements and pathogenic. In mammals, these genomic repeats would recruit the Polycomb/Trithorax proteins (reviewed by Casa and Gabellini, 2012) essential for cell identity and differentiation. Again, at an individual gene level, Polycomb proteins assist transcription factors for gene regulation. At the upper cellular scale, they (super)-structure the cell into compartments and convey information between them.

The Methyl-CpG-Binding-Protein-2 (Della Ragione et al., 2012) is another transcriptional auxiliary which is capable of oligomerization, DNA bridging and condensation, inducing drastic modifications of chromatin topology. Surprisingly, it is specifically over-expressed in neurons, in stoechiometric amounts with histone H1, and competes with H1 binding to nucleosomes. This neuronal chromatin plasticity is questioned in RETT syndrome, a neurodevelopment disorder with transient autistic features due to a defective MeCP2 protein, reversible in mice.

MeCP2 is also pluri-competent: it silences genes through preferential binding to methylated CpG dinucleotides *in vivo*, represses and activates genes independent of methylation, and is involved in RNA splicing.

TFIIIC is the last-born of genome-wide organizers. First known as a compound of basal RNA polymerase III machinery, it also binds separately to wide-spread sites on genome (ETC, COC, others). At a global level, it takes part in long-range action and high-order structures, is an enhancer blocker and counteracts the spreading of heterochromatin. At an individual gene level, this is a repressor of RNA polymerase II transcription, with several features of a prokaryotic factor, narrowing the frontier between prokaryotes and eukaryotes (Amouyal, 2012).

Clearly, genomic architecture and its influence on genetic expression still deserve further investigation. Also, the picture of a regulator is not complete if it is not traced throughout developmental or environmental changes, like CTCF in embryonic and fetal germ cells at the Igf2/H19 locus to specify its role in the setting-up of imprinting (Singh et al., 2012). Last, like emphasized by several articles of this volume, it is difficult to restrict some factors to a unique task. Thus, what some factors might do specifically with respect to genetic expression, other cellular factors might as well do it less specifically. In case of chromosomal looping for instance, any connection between two distant genomic sites might favor or disfavor specific enhancer-promoter interactions, generating a global network of connections at a given time, in a given cell line and a given environment, that the future will specify.
